# Role of redox-inactive metals in controlling the redox potential of heterometallic manganese–oxido clusters

**DOI:** 10.1007/s11120-021-00846-y

**Published:** 2021-05-28

**Authors:** Keisuke Saito, Minesato Nakagawa, Manoj Mandal, Hiroshi Ishikita

**Affiliations:** 1 Department of Applied Chemistry, Graduate School of Engineering, The University of Tokyo, 7-3-1 Hongo, Bunkyo-ku, Tokyo, 113-8654 Japan; 2 Research Center for Advanced Science and Technology, The University of Tokyo, 4-6-1 Komaba, Meguro-ku, Tokyo, 153-8904 Japan

**Keywords:** Water-splitting enzyme, Highest occupied molecular orbital, Density functional theory, Redox potential shift, Artificial Mn clusters, Oxygen-evolving center

## Abstract

Photosystem II (PSII) contains Ca^2+^, which is essential to the oxygen-evolving activity of the catalytic Mn_4_CaO_5_ complex. Replacement of Ca^2+^ with other redox-inactive metals results in a loss/decrease of oxygen-evolving activity. To investigate the role of Ca^2+^ in this catalytic reaction, we investigate artificial Mn_3_[M]O_2_ clusters redox-inactive metals  [M] ([M]  = Mg^2+^, Ca^2+^, Zn^2+^, Sr^2+^, and Y^3+^), which were synthesized by Tsui et al. (Nat Chem 5:293, 2013). The experimentally measured redox potentials (*E*_m_) of these clusters are best described by the energy of their highest occupied molecular orbitals. Quantum chemical calculations showed that the valence of metals predominantly affects *E*_m_(Mn^III/IV^), whereas the ionic radius of metals affects *E*_m_(Mn^III/IV^) only slightly.

**Supplementary Information:**

The online version contains supplementary material available at 10.1007/s11120-021-00846-y.

## Introduction

**Fig. 1:**
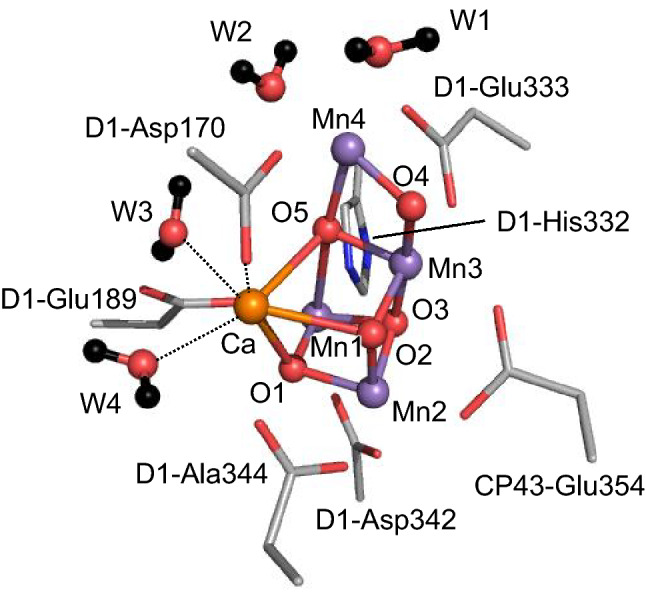
Structure of the Mn_4_CaO_5_ cluster of PSII. Dotted lines indicate ligations to Ca^2+^

Plants, algae, and cyanobacteria use the water-splitting enzyme photosystem II (PSII) for oxygen evolution. The oxygen evolution proceeds at the oxygen-evolving center, the Mn_4_CaO_5_ cluster. The cluster consists of a distorted cubane [Mn1, Mn2, Mn3, four oxygen atoms, and Ca^2+^] and “dangling” Mn4 (Fig. 1) (Umena et al. 2011). The Mn_4_CaO_5_ cluster has two ligand water molecules, W1 and W2, at the Mn4 site and another two ligand water molecules, W3 and W4, at the Ca^2+^ site (Fig. 1). The catalytic cycle moves through a series of oxidation states, denoted as S_*n*_ (*n* = 0, 1, 2, and 3). As electron transfer occurs, S_*n*_ increases. During the catalytic cycle, four electrons from two of the substrate water molecules are removed, and O_2_ evolves in the S_3_ to S_0_ transition (Shen 2015; Cardona and Rutherford 2019).

In the Mn_4_CaO_5_ cluster, a redox-inactive Ca^2+^ is essential for the oxygen evolution activity, as oxygen is not evolved when Ca^2+^ is removed (Ono and Inoue 1988) or replaced with Dy^3+^, Cu^2+^, Cd^2+^ (Lee et al. 2007), K^+^, Rb^+^, and Cs^+^ (Ono et al. 2001). The Mn_4_SrO_5_ cluster can evolve oxygen but the activity is lower than that of the native Mn_4_CaO_5_ cluster (Yachandra and Yano 2011). Koua et al. identified that the distance between Sr^2+^ and W3 (2.6 Å) was longer than that between Ca^2+^ and W3 (2.4 Å) (Koua et al. 2013) and proposed that the long Sr^2+^⋯W3 distance contributed to the decrease in the activity upon replacement of Ca^2+^ with Sr^2+^.

It was speculated that Ca^2+^ might be responsible for the distorted cubane structure of the Mn_4_CaO_5_ cluster (Kawakami et al. 2011). However, the removal of Ca^2+^ does not alter the Mn_3_CaO_4_ cubane structure (Saito and Ishikita 2014; Siegbahn 2014, 2017), as suggested by the extended X-ray absorption fine structure (EXAFS) and the electron paramagnetic resonance (EPR) measurements (Latimer et al. 1998; Yachandra and Yano 2011; Lohmiller et al. 2012). Note that the Jahn–Teller distortion for Mn(III) ions can be affected by Ca^2+^ (Yamaguchi et al. 2013). When Ca^2+^ is removed, the rearrangement of water molecules in the hydrogen-bond (H-bond) network of the redox-active tyrosine (TyrZ) is observed (Saito and Ishikita 2014; Saito et al. 2020a). TyrZ is involved in electron transfer from the Mn_4_CaO_5_ cluster to the reaction center chlorophyll P_D1_. The rearrangement of the H-bond network increases its redox potential (*E*_m_(TyrZ)) by ~ 300 mV and inhibits the formation of the downhill electron transfer pathway from the Mn_4_CaO_5_ cluster via TyrZ to P_D1_ (Saito et al. 2020a). Thus, Ca^2+^ is essential in both maintaining the H-bond network and optimizing electron transfer. The role of Ca^2+^ as the water binding site can be substituted with H_3_O^+^: recent theoretical studies showed that the H-bond network, including the low-barrier H-bond between TyrZ and D1-His190, remains unaltered upon the replacement of Ca^2+^ with H_3_O^+^ (Saito et al. 2020a).

Ca^2+^ is a prerequisite for the low-barrier H-bond between W1 and D1-Asp61: they form a low-barrier H-bond in native PSII (Kawashima et al. 2018b; Saito et al. 2020a), whereas they cannot form in the absence of Ca^2+^ (Saito et al. 2020a). That is, Ca^2+^ decreases p*K*_a_(W1) electrostatically to a level of p*K*_a_(D1-Asp61) in native PSII, thus forming the low-barrier H-bond and facilitating proton transfer from W1 to D1-Asp61.

So far, the role of the Ca^2+^ can be summarized as follows: (i) maintaining the TyrZ H-bond network (Saito et al. 2011, 2020a; Kawashima et al. 2018a), including the low-barrier H-bond between TyrZ and D1-His190 (Kawashima et al. 2018b; Saito et al. 2020a); (ii) optimizing *E*_m_(TyrZ) in the electron transfer cascade (Saito et al. 2020a); and (iii) electrostatically decreasing p*K*_a_(W1) and facilitating proton transfer via the low-barrier H-bond with D1-Asp61 (Saito et al. 2020a).

**Fig. 2:**
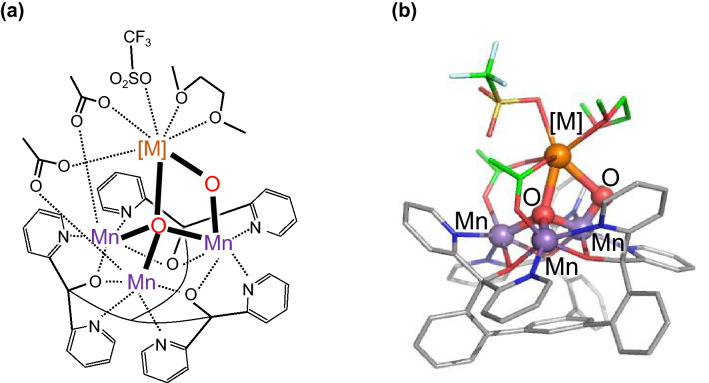
Structure of the Mn_3_[M]O_2_ cluster for [M] = Ca^2+^, Sr^2+^, and Y^3+^ (Tsui et al. 2013) **a** Chemical structure. **b** Three-dimensional structure. For the detail of ligands, see Table S1

Althogh it was proposed that Ca^2+^ might electrostatically affect the properties of the cluster (e.g., p*K*_a_ and *E*_m_) (McEvoy and Brudvig 2006), the replacements of Ca^2+^ with H_2_O and H_3_O^+^ lead to different *E*_m_(Mn^III/IV^) values due to different H-bond patterns in PSII (Saito et al. 2020a). Artificial Mn clusters with redox-inactive metals (Zhang et al. 2015; Mukherjee et al. 2012; Tsui et al. 2013; Kanady et al. 2013; Tsui and Agapie 2013; Lin et al. 2015) may serve as reference model systems since the corresponding H-bond network is absent. Tsui et al. synthesized artificial Mn_3_[M]O_2_ and clusters with redox-inactive metals [M] ([M] = Mg^+^, Ca^2+^, Zn^2+^, Sr^2+^, and Y^3+^) (Fig. 2), showing that *E*_m_(Mn^III/IV^) depends on the Lewis acidity of [M] (i.e., the p*K*_a_ of aqua complexes of [M]) (Tsui et al. 2013). A similar correlation between the ligand-to-metal charge transfer energy (related to *E*_m_) and the Lewis acidity has also been reported in the Fe and Mn complexes (Bang et al. 2014; Krewald et al. 2016).

*E*_m_ can be calculated as the free energy difference between the oxidized and reduced states, including the entropic effect of the solvent (Marenich et al. 2014; Pitari et al. 2015; Amin et al. 2013; Krewald et al. 2016). *E*_m_ also correlates with the ionization potential as shown for various complexes, including Mn complexes (Marenich et al. 2014; Krewald et al. 2016). The ionization potential can be regarded as the free energy difference between the oxidized and the reduced states when the reorganization effect upon the redox reaction (including the electronic relaxation, the solvent reorganization, and the structural change of the molecule) is neglective. As the ionization potential (or the electronic affinity) is correlated with the energy levels of the lowest unoccupied molecular orbital (LUMO) and the highest occupied molecular orbital (HOMO) in density functional theory (DFT) (Kohn–Sham orbital) (Zhang and Musgrave 2007), *E*_m_ should be calculated based on the HOMO or LUMO energy calculated using DFT. An electron releases from the HOMO upon oxidation, whereas an electron enters the LUMO upon reduction. Thus, the HOMO energy corresponds to the potential for one-electron oxidation, and the LUMO energy corresponds to the potential for one-electron reduction. When the redox reaction is reversible, the midpoint potential *E*_m_ is located at the midpoint between the oxidation and reduction potentials, i.e., *E*_m_ and the two potentials have the same tendency. Indeed, the *E*_m_ of quinones can be determined based on the LUMO energy (Ishikita and Saito 2020) as accurately as the free energy difference (Kishi et al. 2017). For complexes that include transition metals, high correlations between the HOMO and/or LUMO energy and the experimentally measured *E*_m_ value were observed [e.g., organic compounds (Mendez-Hernandez et al. 2013), β-diketones complexes, including Mn and Fe (Conradie 2015), and FeCo proteins (Dance 2006)]. For the natural Mn_4_CaO_5_ cluster in the PSII protein environment, *E*_m_ can be determined based on the HOMO energy (Mandal et al. 2020; Saito et al. 2020a, b). Here we calculate the *E*_m_ values of the artificial clusters (Tsui et al. 2013; Tsui and Agapie 2013) based on the HOMO energy and explain how the redox-inactive metal affects *E*_m_(Mn^III/IV^).

## Computational details

The crystal structures of synthetic Mn_3_[M]O_2_ clusters ([M] = Na^+^, Sr^2+^, Ca^2+^, Zn^2+^, and Y^3+^) (Tsui et al. 2013) and Mn_3_[M]O_4_ clusters ([M] = Sr^2+^, Ca^2+^, Zn^2+^, Mn^3+^, Sc^3+^ and Y^3+^) (Tsui and Agapie 2013) were used as the basis for geometry optimization using unrestricted DFT (UDFT), with the B3LYP functional and LACVP* basis set (for optimized structures, see Supporting Information). For efficiency, the cluster was considered to comprise ferromagnetically coupled Mn atoms, (Tsui et al. 2013) where the total spin, *S*, was 12/2 for the Mn_3_[M]O_2_ cluster and 10/2 for the Mn_3_[M]O_4_ cluster. We note that, in the calculation of the native Mn_4_CaO_5_ of PSII, the difference in *S* (e.g., *S* = 1/2 in S_2_ (Zimmermann and Rutherford 1986), high, low, ferromagnetic, and antiferromagnetic) did not affect the (i) resulting geometry (Ames et al. 2011; Isobe et al. 2012), (ii) potential energy profile of proton transfer (Kawashima et al. 2018b), (iii) redox potential of each Mn site (Mandal et al. 2020), or (iv) p*K*_a_ values of ligand water molecules W1–W4 (Saito et al. 2020c). The resulting oxidation states for three Mn atoms were Mn(III)_3_ and Mn(IV)Mn(III)_2_ for the Mn_3_[M]O_2_ and the Mn_3_[M]O_4_ clusters, respectively (for atomic spin density, see Table S2). *E*_m_(Mn1^III/IV^) was calculated from the HOMO energies, since the value of *E*_m_ for one-electron oxidation is correlated with the energy of the highest occupied molecular orbital (HOMO) (Mendez-Hernandez et al. 2013; Igarashi and Seefeldt 2003; Mandal et al. 2020). Using the optimized geometries in vacuum, the HOMO energy (*E*_HOMO_) was calculated in dichloromethane (CH_2_Cl_2_, dielectric constant 8.93) using the polarizable continuum model (PCM). All calculations were performed with Jaguar program [Schrödinger, LLC, 2012, New York]. The initial-guess wavefunctions were obtained using the ligand field theory (Vacek et al. 1999) implemented in the Jaguar program.

## Results and discussion

**Fig. 3:**
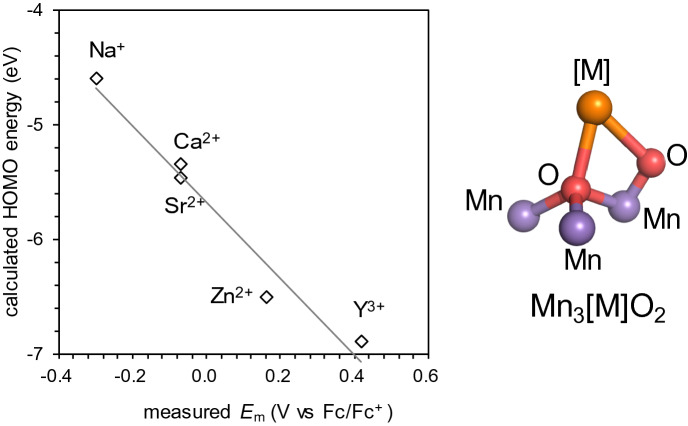
Experimentally measured *E*_m_(Mn^III/IV^) values in CH_2_Cl_2_ (Tsui and Agapie 2013) and calculated HOMO energy levels (*E*_HOMO_) of the Mn_3_[M]O_2_ clusters. The coefficient of determination (*R*^2^) is 0.96

The calculated *E*_HOMO_ values for the artificial Mn_3_[M]O_2_ clusters ([M] = Na^+^, Sr^2+^, Ca^2+^, Zn^2+^, and Y^3+^) show a correlation with the experimentally measured *E*_m_(Mn^III/IV^) values (Fig. 3) in CH_2_Cl_2_ and are best fitted to Eq. (1).

1 $$E_{{\rm{m}}} \;{\text{(V}}\;{\text{vs}}{.}\;{\text{Fc/Fc}}^{ + } {) = } -{0}{{.302}}\;E_{{{\rm{HOMO}}}} \;({\text{eV}}) -1.710,$$

where Fc/Fc+ denotes ferrocene electrode. A similar correlation is also observed in the Mn_3_[M]O_4_ cubane clusters ([M] = Sr^2+^, Ca^2+^, Zn^2+^, Mn^3+^, Sc^3+^, and Y^3+^) (Tsui and Agapie 2013) (Fig. S1). The coefficient of −0.302 in Eq. (1) is the conversion factor from MO energy to *E*_m_, which may be associated with the solvation effect (Schmidt am Busch and Knapp 2005), whereas the offset of −1.710 V is associated with a difference between the absolute electrode potential and the Fc/Fc^+^ electrode potential and liquid junction potential (Kishi et al. 2017). These factors depend on the size and the net charge of the QM system, the solvent, and the reference electrode (e.g., see the caption of Fig. S1). Thus, Eq. (1) is applicable only to similar molecular groups (e.g., artificial Mn_3_[M]O_2_ clusters).

**Fig. 4:**
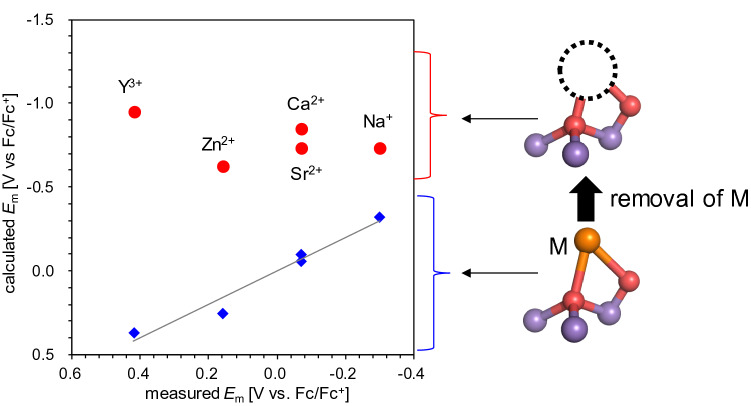
Calculated *E*_m_(Mn^III/IV^) values of the Mn_3_[M]O_2_ clusters (blue diamonds) and calculated *E*_m_(Mn^III/IV^) values of the metal-removed Mn_3_O_2_ clusters (red circles) plotted with experimentally measured *E*_m_(Mn^III/IV^) values in CH_2_Cl_2_ (Tsui and Agapie 2013)

Using Eq. (1), *E*_HOMO_ can be converted to *E*_m_. The calculated *E*_m_ values correlate with the experimentally measured *E*_m_ values (Fig. 4, blue diamonds). In the Mn_3_[M]O_2_ clusters synthesized by Tsui, each [M] has different ligand groups (Table S1). Accordingly, the absolute *E*_m_ values are affected by [M] and the ligand groups. To evaluate the direct influence of electrostatic and the van der Waals interactions with [M] on the *E*_m_ of the Mn_3_[M]O_2_ cluster, the metal [M] was removed from the geometry-optimized Mn_3_[M]O_2_ cluster. The calculated *E*_m_ values for the metal-removed clusters do not correlate with the experimentally measured *E*_m_ values (Fig. 4, red circles)._._

**Fig. 5:**
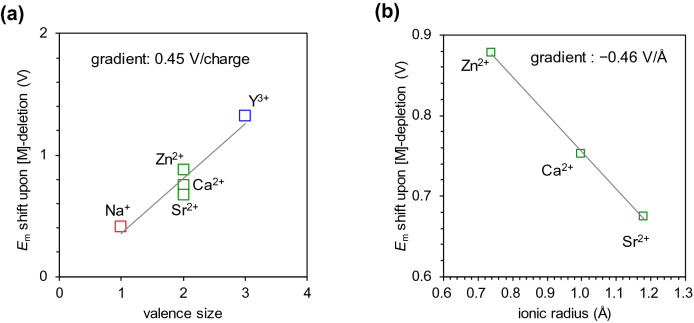
Shift in *E*_m_ upon the removal of [M] ([M] = Na^+ ^(red), Sr^2+^, Ca^2+^, Zn^2+ ^(green), and Y^3+ ^(blue)) **a** with respect to the valence (*R*^2^ = 0.93) and **b** with respect to the ionic radius (Shannon 1976) of [M] ([M] = Sr^2+^, Ca^2+^, and Zn^2+^) (*R*^2^ = 0.99)

The removal of Y^3+^ resulted in an increase of 1.5 V in *E*_m_, whereas the removal of Na^+^ resulted in an increase of 0.5 V in *E*_m_ (Fig. 5a). These results suggest that the valence of [M] is the main factor determining *E*_m_. In addition, the removal of a metal with a large radius (e.g., Sr^2+^) resulted in a large increase in *E*_m_, whereas the removal of a metal with a small radius (e.g., Zn^2+^) resulted in a small increase in *E*_m_ (Fig. 5b). For metals with the same valence (e.g., Sr^2+^, Ca^2+^, and Zn^2+^), the difference in *E*_m_ can be explained by the difference in the ionic radius of the redox-inactive metal [M]. As the radius of [M] increases, the distance between [M]^2+^ and Mn increases, leading to weak electrostatic interactions between Mn and [M]^2+^. Thus, of the [M]^2+^ metals, [M] with large radii have a smaller influence on *E*_m_. The effect that the ionic radius has on the difference in *E*_m_ can be explained in terms of the Lewis acidity of [M] because the Lewis acidity decreases with an increase in the ionic radius (Lin et al. 2015).

In PSII, *E*_m_(Mn1^III/IV^) of the Mn_4_CaO_5_ cluster changes by only ~40 mV even upon the replacement ion of Ca^2+^ with H_3_O^+^ irrespective of the loss of a +1 charge (Saito et al. 2020a). In contrast, *E*_m_(Mn^III/IV^) of the Mn_3_[M]O_2_ cluster changes by 450 mV upon the loss of a +1 charge (Fig. 5a). These indicate that the protein environment including the H-bond network (e.g., D1-Asp61, TyrZ, D1-His190, and water molecules) plays a key role in determining the *E*_m_(Mn_4_CaO_5_) in PSII.

In summary, the quantum-chemically calculated HOMO energies of artificial Mn_3_[M]O_2_ clusters ([M] = Na^+^, Sr^2+^, Ca^2+^, Zn^2+^, and Y^3+^) correlate with the experimentally measured *E*_m_(Mn^III/IV^) values (Fig. 3). The *E*_m_ calculation for the metal-deleted Mn_3_O_2_ clusters shows that the valence of [M] predominantly affects *E*_m_ (Fig. 5a), whereas the ionic radius of [M] affects *E*_m_ only slightly (Fig. 5b).

## Supplementary Information

Below is the link to the electronic supplementary material.

Supplementary file1 (PDF 200 kb)

Supplementary file3 (PDB 55 kb)

Supplementary file4 (PDB 67 kb)
