# Supplementary material for: Role of redox-inactive metals in controlling the redox potential of heterometallic manganese–oxido clusters
Source: Photosynth Res. 2021 May 28;148(3):153–9. doi: 10.1007/s11120-021-00846-y (PMC8292285; doi:10.1007/s11120-021-00846-y)
Supplement: Supplementary file 1 — Supplementary file1 (PDF 200 kb) [file 11120_2021_846_MOESM1_ESM.pdf]

Supplementary information

Role of redox-inactive metals in controlling the redox potential of heterometallic manganese-oxido clusters

Keisuke Saito[1,2], Minesato Nakagawa[1], Manoj Mandal[2], and Hiroshi Ishikita[1,2*]*

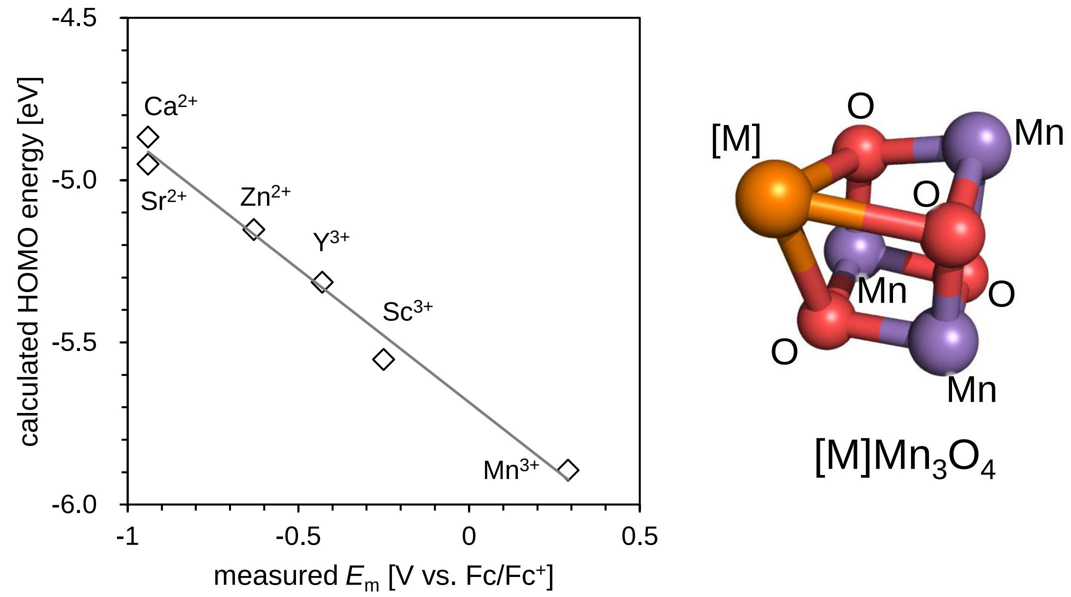

Figure S1. Experimentally measured E_m (Mn^{III} Mn^{IV}_2/Mn^{IV}_3) values in solvent mixed with dichloromethane:dimethoxyethane = 10:1 and calculated HOMO energy levels (E_{HOMO}) of the $Mn_3[M]O_4$ clusters (Tsui and Agapie 2013). The coefficient of determination (R^2) = 0.99. The experimentally measured E_m($Mn^{III/IV}$) values are best fitted to E_m (V vs. Fc/Fc$^+$) = –1.22 E_{HOMO} (eV) – 6.93, which has quite different coefficients in comparison with eq. (1), because of the different solvent and charge of the system.

Table S1. Ligands of the cluster (Tsui et al. 2013).

cluster	Ligands[a]
Mn_3NaO_2	CH_3COO^{-}×3, L
Mn_3SrO_2	CH_3COO^{-}×2, $CF_3SO_3^{-}$, DME, L
Mn_3CaO_2	CH_3COO^{-}×2, $CF_3SO_3^{-}$, DME, L
Mn_3ZnO_2	CH_3COO^{-}×2, CH_3CN, L
Mn_3YO_2	CH_3COO^{-}×2, $CF_3SO_3^{-}$, DME, L

[a] DME: 1,2-dimethoxyethane;
L: 1,3,5-tris(2-di(2′-pyridyl)hydroxymethylphenyl)benzene

Table S2. Mulliken atomic spin densities of Mn atoms.

	Mn_3NaO_2	Mn_3SrO_2	Mn_3CaO_2	Mn_3ZnO_2	Mn_3YO_2
**Mn1[a]**	3.71	3.74	3.74	3.79	3.82
**Mn2A[a]**	3.91	3.90	3.89	3.87	3.90
**Mn2B[a]**	3.86	3.85	3.85	3.84	3.86

	Mn_3SrO_4	Mn_3ZnO_4	Mn_3CaO_4	Mn_4O_4	Mn_3ScO_2	Mn_3YO_2
**Mn1[b]**	2.93	2.93	2.91	3.84	2.92	3.88
**Mn2[b]**	3.87	3.87	2.93	2.92	3.88	2.93
**Mn3[b]**	2.91	2.91	3.87	2.87	2.91	2.91
**Mn4**	-	-	-	3.86	-	-

[a] For the atomic notation, see Figure S2. Note that Mn2A and Mn2B are identical.
[b] Note that Mn1, Mn2, and Mn3 are identical.

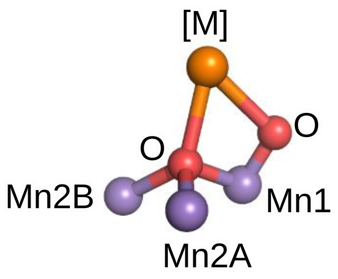

Figure S2. Atomic notation of Mn in the Mn3[M]O2 cluster.

Reference

Tsui EY, Agapie T (2013) Reduction potentials of heterometallic manganese-oxido cubane complexes modulated by redox-inactive metals. Proc Natl Acad Sci U S A 110 (25):10084-10088

Tsui EY, Tran R, Yano J, Agapie T (2013) Redox-inactive metals modulate the reduction potential in heterometallic manganese-oxido clusters. Nat Chem 5 (4):293-299
